# Heterogeneity in COVID-19 infection among older persons in South Africa: Evidence from national surveillance data

**DOI:** 10.3389/fpubh.2023.1009309

**Published:** 2023-03-16

**Authors:** Nada Abdelatif, Inbarani Naidoo, Shanaaz Dunn, Mikateko Mazinu, Zaynab Essack, Candice Groenewald, Pranitha Maharaj, Nokukhanya Msomi, Tarylee Reddy, Benjamin Roberts, Khangelani Zuma

**Affiliations:** ^1^Biostatistics Research Unit, South African Medical Research Council, Cape Town, South Africa; ^2^Centre for Community Based Research, Human and Social Capabilities Division, Human Sciences Research Council, Durban, South Africa; ^3^School of Built Environment and Development Studies, University of KwaZulu-Natal, Durban, South Africa; ^4^Centre for Community Based Research, Human and Social Capabilities Division, Human Sciences Research Council, Pietermaritzburg, South Africa; ^5^Honorary Research Fellow, School of Law, and Honorary Research Associate, Centre for the AIDS Programme of Research in South Africa, University of KwaZulu-Natal, Durban, South Africa; ^6^Honorary Research Associate, Rhodes University, Grahamstown, South Africa; ^7^Discipline of Virology, University of KwaZulu-Natal and National Health Laboratory Services, Durban, South Africa; ^8^Biostatistics Research Unit, South African Medical Research Council, Durban, South Africa; ^9^Developmental, Capable and Ethical State Division, Human Sciences Research Council, Durban, South Africa; ^10^Human and Social Capabilities Division, Human Sciences Research Council, Pretoria, South Africa; ^11^Wits School of Public Health, University of Witwatersrand, Johannesburg, South Africa

**Keywords:** older adult people, heterogeneity, COVID-19, surveillance, South Africa

## Abstract

**Background:**

The 2021 World Health Organization study on the impact of COVID-19 on older people (≥60 years) in the African region highlighted the difficulties they faced as the virus spread across borders and dominated the way of life. These difficulties included disruptions to both essential health care services and social support, as well as disconnections from family and friends. Among those who contracted COVID-19, the risks of severe illness, complications, and mortality were highest among near-old and older persons.

**Objective:**

Recognizing that older persons are a diverse group including younger- and older-aged individuals, a study was conducted to track the epidemic among near-old (50–59 years) and older persons (≥60 years) in South Africa covering the 2 years since the epidemic emerged.

**Methods:**

Using a quantitative secondary research approach, data for near-old and older persons were extracted for comparative purposes. COVID-19 surveillance outcomes (confirmed cases, hospitalizations, and deaths) and vaccination data were compiled up to March 5th, 2022. COVID-19 surveillance outcomes were plotted by epidemiological week and epidemic waves to visualize the overall growth and trajectory of the epidemic. Means for each age-group and by COVID-19 waves, together with age-specific rates, were calculated.

**Results:**

Average numbers of new COVID-19 confirmed cases and hospitalizations were highest among people aged 50–59- and 60–69-years. However, average age-specific infection rates showed that people aged 50–59 years and ≥80 years were most vulnerable to contracting COVID-19. Age-specific hospitalization and death rates increased, with people aged ≥ 70 years most affected. The number of people vaccinated was slightly higher among people aged 50–59 years before Wave Three and during Wave Four, but higher among people aged ≥ 60 years during Wave Three. The findings suggest that uptake of vaccinations stagnated prior to and during Wave Four for both age groups.

**Discussion:**

Health promotion messages and COVID-19 epidemiological surveillance and monitoring are still needed, particularly for older persons living in congregate residential and care facilities. Prompt health-seeking should be encouraged, including testing and diagnosis as well as taking up vaccines and boosters, particularly for high-risk older persons.

## 1. Introduction

Africa is described as being the youngest of the five world regions, due to having the lowest proportion of people aged ≥ 60 years of the total population (1). However, reflecting on the size of the older persons population within Africa shows a projected tripling in the growth rate from 74.4 million in 2020 to 235 million in 2050 (1). South Africa has been ranked among the top six African countries with the highest older persons population of people aged ≥ 60 years (1). In South Africa there are ~5.4 million people aged ≥ 60 years; comprising 59.8% of people aged 60–69 years, 29.8% of people aged 70–79 years and 10.5% of people aged ≥ 80 years (2). It has therefore become increasingly relevant to focus on the health and wellbeing of people living to older ages (3). In many African countries, care for older people is the responsibility of family members, with little or no government support (4–6). For the work presented here, an older person refers to any person aged ≥ 60 years (7, 8). This analyses included people aged 50–59 years, to represent the “near-old” age group who will augment the rapidly growing proportion of older persons.

The emergence and spread of COVID-19 brought uncertainty, mental and financial hardship, and far-reaching consequences for infected and affected older persons. Globally COVID-19 has disproportionately affected older people, both directly and indirectly. The health and wellbeing of older persons living in Africa lag behind those of other world regions (9), as do the health systems where they live (10). A 2020 United Nations policy brief outlined the negative impacts of COVID-19 among older persons (11). Particular challenges included reduced access to health care services for health outcomes unrelated to COVID-19, forms of discrimination due to being aged, isolation due to social distancing, as well as neglect and abuse in some institutional facilities (11). Furthermore, a WHO report on the impact of COVID-19 on older people in the African region highlighted the insufficient focus on aging, with limited accessible age and sex disaggregated data for older persons (9). The paucity of age disaggregated data for older persons limits the extension of knowledge and inclusivity of COVID-19 interventions targeting older persons' specific needs.

South Africa had the highest incidence of confirmed COVID-19 cases in the African region and experienced five epidemic waves by May 2022 (12). The First Wave, was driven by an ancestral variant in mid-2020, followed by the Second Wave at the end of 2020, driven by the Beta variant, which saw a relatively higher number of documented infections (13). The Third Wave in mid-2021, driven by the Delta variant, was the most significant in terms of confirmed cases and mortality, and led to an overwhelmed healthcare system. The Omicron variant became the dominant variant in December 2021, driving the Fourth Wave in the country. Although highly transmissible with the highest number of confirmed cases seen to date, it resulted in milder disease in the population. South Africa entered the Fifth Wave in May 2022, driven by two sub-lineages of the Omicron variant. Although the trends in confirmed COVID-19 cases, hospitalizations and deaths have been reported, most of these studies have focused on the differences between younger and older people (11, 14). Comparison of outcomes in hospital admissions have been described for people aged ≥ 50 years in 10-year intervals (15). However, specific COVID-19 heterogeneity between near-old (50–59 years) and older people (≥60 years), particularly retrospectively, and over a time frame that is longer than a 1-year period have been unexplored to our knowledge.

Older persons in South Africa, particularly, have several challenges, including a high proportion with no formal education, approximately one fifth lived below the food poverty line in 2015, and a large proportion of households were headed by female older persons (2) who support multiple dependents. In addition, ailments such as hypertension, diabetes, arthritis, heart disease, asthma, high cholesterol levels and stroke are prevalent in older persons (2). Over half of people aged ≥ 60 years reported having hypertension, 10% of people aged ≥ 70 years were diagnosed with heart disease, and more than 17% reported having cancer (16). The severity of COVID-19 disease disproportionately affected older persons compared to younger people, evidenced by higher positivity rates and increased rates of hospitalizations and deaths compared to their younger counterparts (9, 17–21). The risk of severe disease increased with the presence of one or more of the co-morbidities mentioned above. To offset the economic and social challenges (22, 23), which were exacerbated during the COVID-19 pandemic, the current situation of older persons warrants consideration to respond to their immediate needs together with provisions for adequate support and protection of the near-old age group.

Weekly epidemiological updates commencing March 2020 were released by the National Institute for Communicable Diseases (NICD) in South Africa, showing the national and provincial trends of confirmed COVID-19 cases by age and sex, together with weekly testing summary reports and hospital surveillance reports from the Data for COVID surveillance system (DATCOV) (24). Thus, the age-related data from the South African Notifiable Medical Conditions Surveillance System (NMCSS) is a valuable and unique resource to explore variations in the epidemic trajectory for near-old and older persons. A review of COVID-19 surveillance efforts in 13 African countries showed that South Africa was the only country to implement nine approaches to surveillance, covering case, hospital and deaths notification, contract tracing, virology, genome, environmental and community aspects (25). This paper aims to explore the differences in COVID-19 surveillance outcomes for near-old persons (50–59 years) and older persons (≥60 years), using publicly accessible surveillance data from the South African national data repository for a 2-year period.

## 2. Ethical approval

The study was approved by the Human Sciences Research Council Ethics Committee (REC) Protocol Number: (REC 3/17/03/21).

## 3. Methods

This study examined COVID-19 confirmed cases, hospitalizations, deaths and vaccination uptake. Publicly available NICD COVID-19 Weekly Epidemiology Briefs and Daily Sentinel Hospital Surveillance reports were used as data sources to manually extract confirmed COVID-19 cases, hospitalizations and deaths for 2020 and 2021. Hospital admissions data contained in these reports were for people admitted for COVID-19 or who had tested positive during their hospital stay. Publicly available age-disaggregated vaccine data were found on the South African National Department of Health COVID-19 Online Resource and News Portal website (https://sacoronavirus.co.za/), which were established as valuable information resources for a broad audience early in the epidemic trajectory (25, 26). Confirmed cases, hospitalizations, deaths and vaccination uptake were extracted and processed for four age-groups (50–59, 60–69, 70–79, and ≥80 years) where possible. Only aggregated data were available and the data sources did not contain any individual level data.

South Africa's first confirmed COVID-19 case was publicly announced on March 5th, 2020. Thereafter, the first available report for confirmed COVID-19 cases contained data from April 27th, 2020 (epidemiological week 17 of 2020). Hospitalization and death data were first available from May 30th, 2020 (epidemiological week 22 of 2020) and vaccination data from February 17th, 2021 (epidemiological week 7 of 2021). All data were extracted up to March 5th, 2022 (epidemiological week 9 of 2022). There were missing data in confirmed cases in the weekly reports from epidemiological weeks 12–26 in 2021. For deaths, seven data points were missing for epidemiological weeks 30–36 in 2020, inclusive. These were estimated using a ratio to estimate the data for people aged 50–59 years. Hospital admissions and death data for epidemiological weeks 40–43 of 2020 for all age groups were not publicly available. No interpolation or imputation were performed on cases, hospitalizations or deaths to fill in the data gaps. The data were retrieved from the online datasets and analyzed as reported and missing information for the target age groups from the NICD reports were not included in this study. Cumulative vaccines administered were provided for people aged 50–59 years and ≥60 years. Hence these data could not be presented in the 10-year age intervals as listed above.

This was a retrospective descriptive epidemiological study using routinely collected surveillance data to describe the differential impacts of COVID-19 on four target age-groups over the first four COVID-19 waves as defined for South Africa (Table 1). Absolute numbers were presented and compared, as well as age-group-specific rates per 1,000 people for cases, hospitalizations, deaths and vaccine outcomes using Statistics South Africa's mid-year population estimates for 2020 (27) and 2021 (2) as the denominators. The numbers of new cases, deaths, and admissions were derived from the cumulative data. The case fatality ratio (CFR) was calculated by dividing the number of confirmed cases by the number of COVID-19 deaths. The number of people unvaccinated (vaccine naïve) for each age group was derived by subtracting the number vaccinated from the population in each age group. Trends over the first four COVID-19 waves were inspected by age-group. Microsoft Excel and Stata Version 15 (StataCorp, 2017, Stata Statistical Software, StataCorp) were used for data processing, generating graphs and rate calculations.

**Table 1:** COVID-19 waves 1–4.

**Wave**	**Variant**	**Epidemiological weeks**	**Dates**
1	D614G (Ancestral)	24–34	June 7, 2020–August 22, 2020
2	Beta	47–5	November 15, 2020–February 6, 2021
3	Delta	19–37	May 9, 2021–September 18, 2021
4	Omicron BA.1/BA2	48–5	November 28, 2021–February 5, 2022

Cumulative COVID-19 testing data were additionally extracted for the period March 1st, 2020 (epidemiological week 10 of 2020) to January 9th, 2022 (epidemiological week 2 of 2022). Although testing was not one of the main outcomes of this analyses since these data were not publicly available for the target age groups, they have been included to illustrate the general testing pattern and test positivity over the period. No further analyses were done using the testing data. The testing summary reports contained the proportion that tested positive among specific age groups (40–59, 60–69 and ≥70 years), and hence were not aligned with our target groups of near-old and older persons age categories.

## 4. Results

### 4.1. Testing and positivity

**Figure 1:**
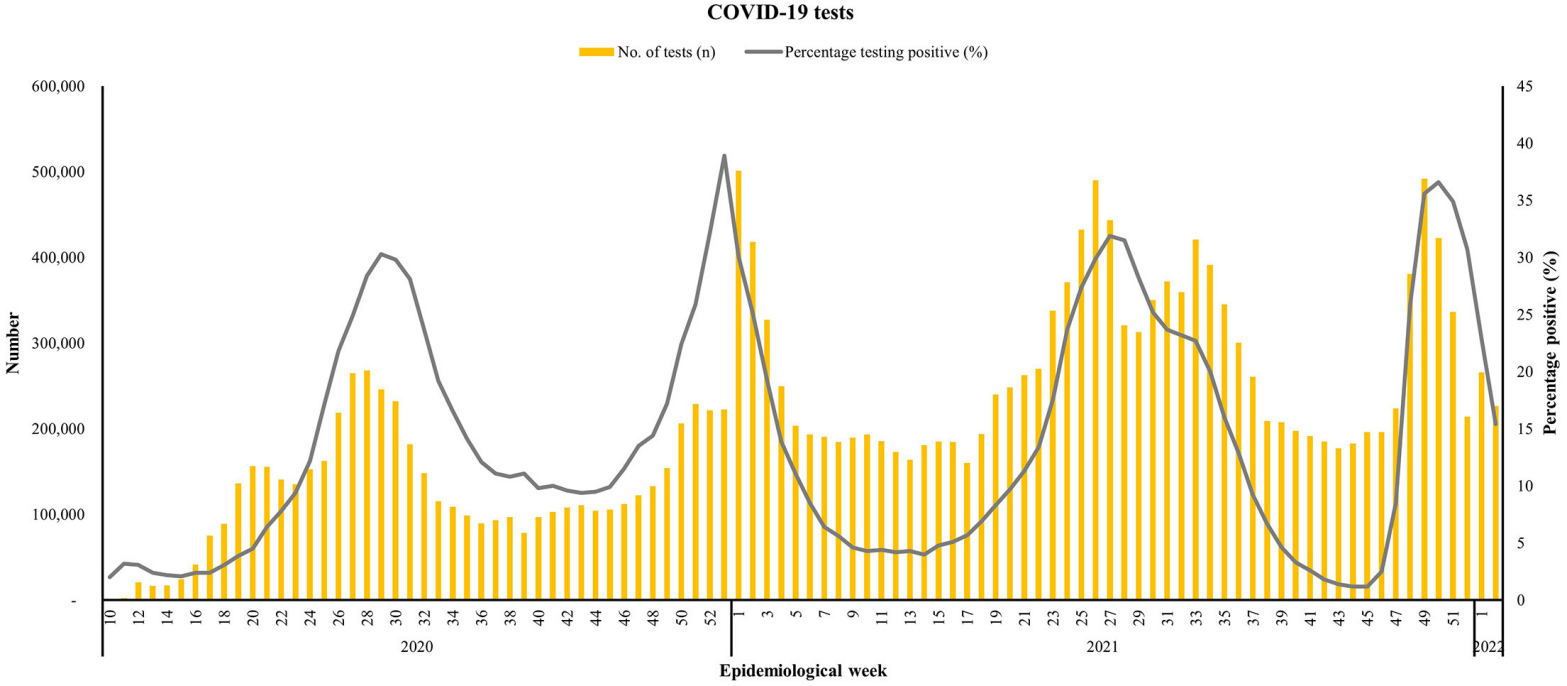
Number of COVID-19 tests conducted for all ages and the percentage that were positive for all ages from March 1st, 2020 to January 9th, 2022, South Africa. Data source: https://www.nicd.ac.za/diseases-a-z-index/disease-index-covid-19/surveillance-reports/weekly-testing-summary/.

The number of confirmed cases was determined from the number of people who took a COVID-19 test, and the results were submitted through the notification system in South Africa. The number of polymerase chain reaction (PCR) tests as well as the test positivity done for all ages are aligned with the timing of the peaks and troughs observed for the COVID-19 waves (Figure 1). However, the percentage testing positive for COVID-19 better reflects transmission of COVID-19, since testing uptake varied and was not taken up by all infectious people in the population, particularly if they were asymptomatic or unaware of an exposure to a confirmed case. The peak test positivity rate (TPR) was similar during Waves One and Three, at ~30%, whereas Wave Two had a TPR of ~40%. Wave Four had a TPR of 37%.

For confirmed COVID-19 cases, hospitalization, deaths and vaccinations, the trends and population adjusted age-specific rates were examined by each epidemic wave, within each target age group and compared among the age groups.

### 4.2. Confirmed cases and age-specific case rates

**Figure 2:**
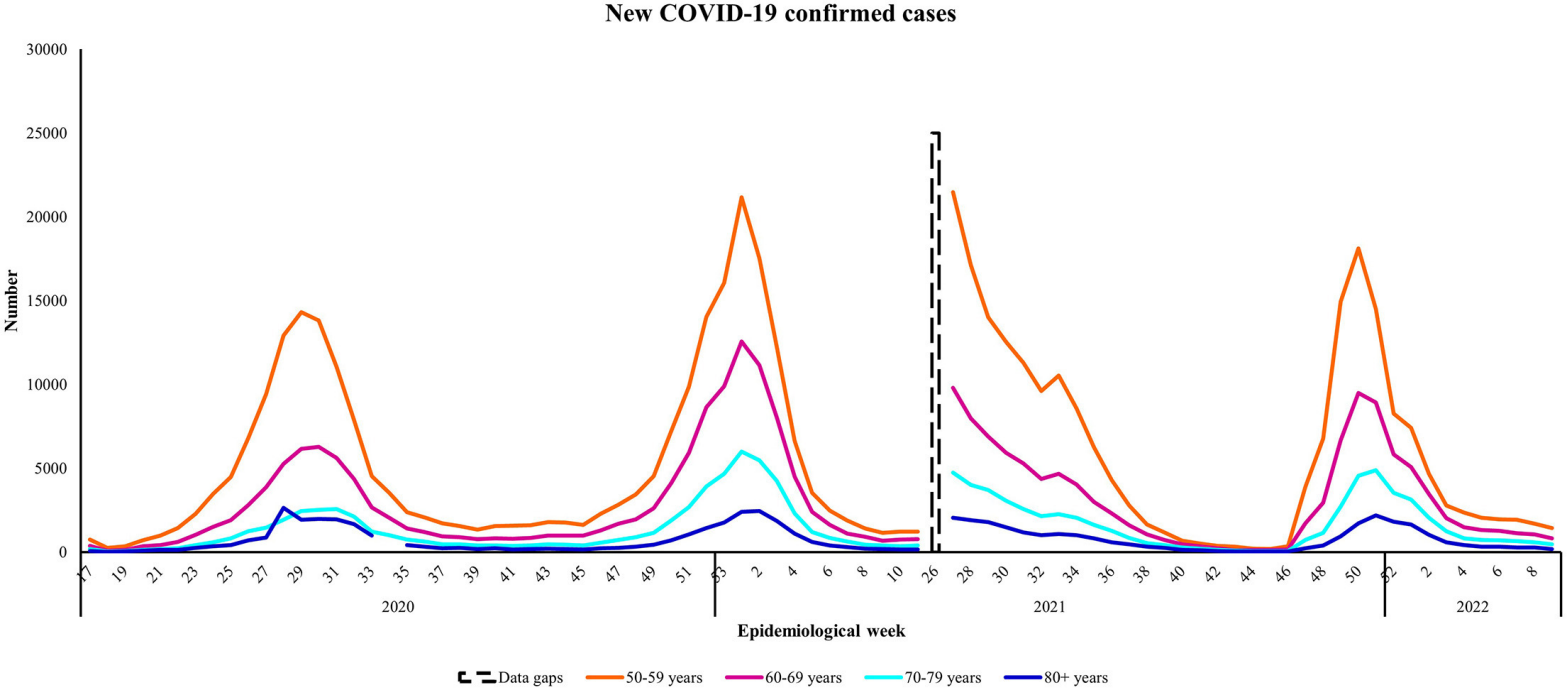
Number of new COVID-19 confirmed cases by age group from April 27th, 2020 to March 5th, 2022, South Africa. Data source: https://www.nicd.ac.za/diseases-a-z-index/disease-index-covid-19/surveillance-reports/weekly-epidemiological-brief/.

Closer inspection of the graphed cases within each target age group shows distinct peaks or waves in COVID-19 cases and revealed differences among the age categories. All waves recorded higher number of cases among people aged 50–59 years followed by people aged 60–69 years of age. For people aged 50–59 years, the highest observed number up until March 5th, 2022 occurred in epidemiological week 27 of 2021, coinciding with Wave Three of the epidemic in the country (Figure 2). By contrast, the highest number of observed cases among people aged 60–69 and 70–79 years occurred during Wave Two. The highest observed number for ≥80 year old people occurred early in the epidemic, during Wave One. Overall, across all four epidemic waves, the highest average number of confirmed COVID-19 cases occurred in the 50–59 year old age group (*N* = 102,958), followed by their counterparts aged 60–69 years (*N* = 54,800) (Table 2).

**Table 2:** Total number of COVID-19 confirmed cases, hospitalizations, deaths, and cumulative number vaccinated^#^ with age-specific rates^*^ within the four COVID-19 waves in South Africa 2020–2022.

**Wave**	**Age group**	**Number of cases (range)**	**Number of hospitalizations (range)**	**Number of deaths (range)**	**Cumulative number of vaccinations^#^ (range)**	**Age-specific case rate (per 1,000 people) (95% CI)**	**Age-specific hospitalization rate (per 1,000 people) (95% CI)**	**Age-specific death rate (per 1,000 people) (95% CI)**	Age-specific vaccination uptake rate^*^(per 1,000 people) (95% CI)
1	50–59	92,282 (3,471–14,326)	12,769 (417–2,657)	2,104 (67–400)	No vaccines available	19.3 (19.05, 19.56)	2.7 (2.43, 3.00)	0.4 (0.16, 0.75)	No vaccines available
	60–69	42,535 (1,515–6,286)	9,097 (275–1,806)	2,296 (74–400)		13.3 (12.98, 13.63)	2.8 (2.47, 3.16)	0.7 (0.40, 1.13)	
	70–79	17,968 (580–2,572)	5,283 (172–960)	1,683 (54–250)		10.9 (10.45, 11.37)	3.2 (2.74, 3.71)	1.0 (0.59, 1.61)	
	80+	13,543 (342–2,648)	3,111 (78–613)	1,217 (44–200)		23.5 (22.79, 24.23)	5.4 (4.63, 6.25)	2.1 (1.40, 3.12)	
2	50–59	119,113 (2,829–21,163)	27,302 (720–5,636)	5,567 (150–908)	No vaccines available	24.9 (24.65, 25.15)	5.7 (5.43, 5.98)	1.2 (0.93, 1.53)	No vaccines available
	60–69	73,499 (1,689–12,559)	24,479 (396–5,380)	7,511 (160–1,244)		22.9 (22.60, 23.21)	7.6 (7.27, 7.94)	2.3 (1.98, 2.67)	
	70–79	35,185 (718–6,004)	14,962 (221–3,261)	5,618 (103–951)		21.4 (20.97, 21.83)	9.1 (8.65, 9.58)	3.4 (2.94, 3.91)	
	80+	14,456 (265–2,461)	6,670 (5–1,554)	2,762 (0–468)		25.0 (24.30, 25.71)	11.6 (10.85, 12.40)	4.8 (4.05, 5.68)	
3	50–59	118,579 (2,794–21,489)	34,067 (521–3,367)	7,939 (77–848)	2,361,366 (2,776–327,397)	24.4 (24.16, 24.65)	7.0 (6.73, 7.28)	1.6 (1.34, 1.90)	485.0 (484.36–485.84)
	60–69	55,920 (1,608–9,811)	35,866 (568–2,537)	9,037 (128–920)	3,426,830 (9,576–411,770)	17.3 (16.99, 17.62)	11.1 (10.78, 11.43)	2.8 (2.47, 3.16)	622.5 (621.99–623.01)
	70–79	28,329 (852–4,747)	20,679 (365–1,864)	7,973 (102–784)		16.9 (16.47, 17.34)	12.4 (11.95, 12.86)	4.8 (4.35, 5.30)	
	80+	13,396 (457–2,063)	11,271 (201–1,058)	5,079 (63–502)		22.5 (21.79, 23.22)	18.9 (18.18, 19.63)	8.5 (7.75, 9.31)	
4	50–59	81,859 (2,049–18,111)	7,344 (249–1,109)	823 (31–186)	726,032 (7,818–48,059)	16.8 (16.54, 17.06)	1.5 (1.23, 1.80)	0.2 (0.03, 0.88)	149.1 (148.28–149.92)
	60–69	47,245 (1,312–9,494)	7,330 (196–1,234)	1,150 (38–273)	413,766 (4,445–29,222)	14.6 (14.28, 14.92)	2.3 (1.97, 2.68)	0.4 (0.14, 1.01)	75.2 (74.40–76.01)
	70–79	24,821 (725–4,881)	6,504 (152–1,125)	1,262 (15–279)		14.8 (14.36, 15.25)	3.9 (3.45, 4.41)	0.8 (0.38, 1.45)	
	80+	11,117 (320–2,202)	4,478 (91–827)	1,056 (13–221)		18.7 (17.98, 19.44)	7.5 (6.75, 8.31)	1.8 (1.09, 2.80)	
Average per age group over all four waves	50–59	102,958 (2,049–21,489)	20,371 (249–5,636)	4,108 (31–908)		21.1 (20.85, 21.35)	4.2 (3.93, 4.49)	0.8 (0.55, 1.13)	
	60–69	54,800 (1,312–12,559)	19,193 (196–5,380)	4,999 (38–1,244)		16.9 (16.59, 17.22)	5.9 (5.57, 6.24)	1.5 (1.18, 1.88)	
	70–79	26,576 (580–6,004)	11,857 (152–3,261)	4,134 (15–951)		15.9 (15.46, 16.35)	7.1 (6.65, 7.58)	2.5 (2.04, 3.01)	
	80+	13,128 (265–2,648)	6,383 (5–1,554)	2,529 (0–502)		22.1 (21.39, 22.82)	10.7 (9.95, 11.49)	4.2 (3.44, 5.05)	

^**#**^Total new number of vaccines administered up to and including wave period.

^*^Using mid-year population estimates for 2020 (27) and 2021 (2).

The age-specific case rates show that people aged ≥ 80 years had the highest burden in all waves followed by their near-old counterparts aged 50–59 years. The exception was for Wave Three, where people aged 50–59 years had a higher case burden compared to all other target age groups (Table 2). Review of the average age-specific infection rates for all four waves suggests that people aged 50–59 years and ≥80 years were most susceptible to acquiring COVID-19 over the 2-year study period.

### 4.3. Hospital admissions and age-specific hospitalization rates

**Figure 3:**
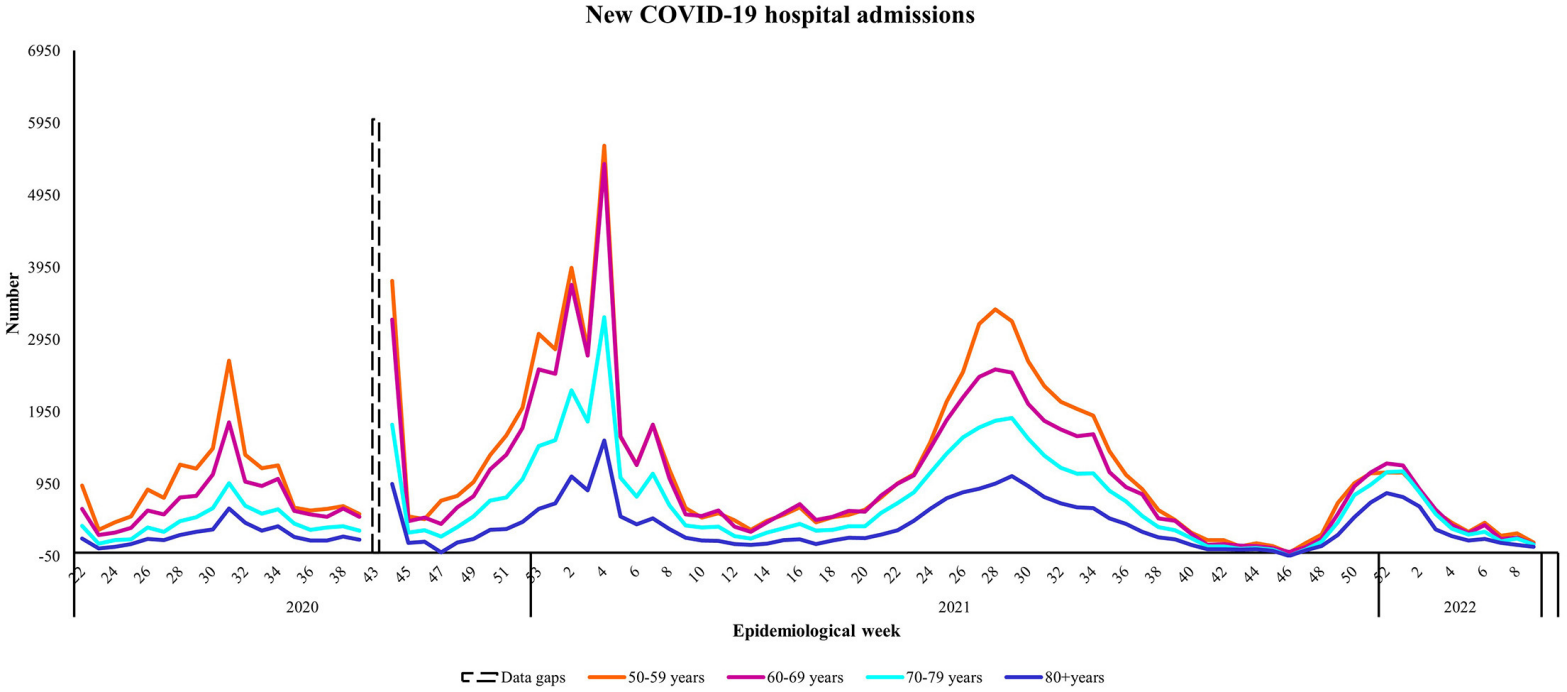
The number of new COVID-19 hospital admissions by age group from May 8th, 2020 to March 5th, 2022, South Africa. Data source: https://www.nicd.ac.za/diseases-a-z-index/disease-index-covid-19/surveillance-reports/daily-hospital-surveillance-datcov-report/.

Overall, across all four epidemic waves, the average number of observed hospital admissions was highest among near-old people aged 50–59 years (*N* = 20,371), followed by people aged 60–69 years (*N* = 19,193) (Table 2). The number of new COVID-19 hospital admissions for all target age groups was highest in the beginning of 2021 during Wave Two (Figure 3). Observed hospitalization numbers were highest among people aged 50–59 and 60–69 years during the first year of the epidemic in South Africa. Wave Three saw a higher observed number of people aged 50–59 years being hospitalized compared to the other older persons, whereas Wave Four had the lowest observed number of hospitalization admissions for all age groups. Overall hospitalization rates were lowest in Wave One when considering the age-specific rates (Table 2), increasing in Waves Two and Three and then decreasing in Wave Four. Hospitalization rates increased almost 2-fold as age increased from 50–59 to ≥80 years in Wave Three. The average age-specific hospitalization rates for all four waves suggests that older persons aged ≥ 70 years were most vulnerable to infections requiring hospitalization over the 2-year study period.

### 4.4. Mortality

**Figure 4:**
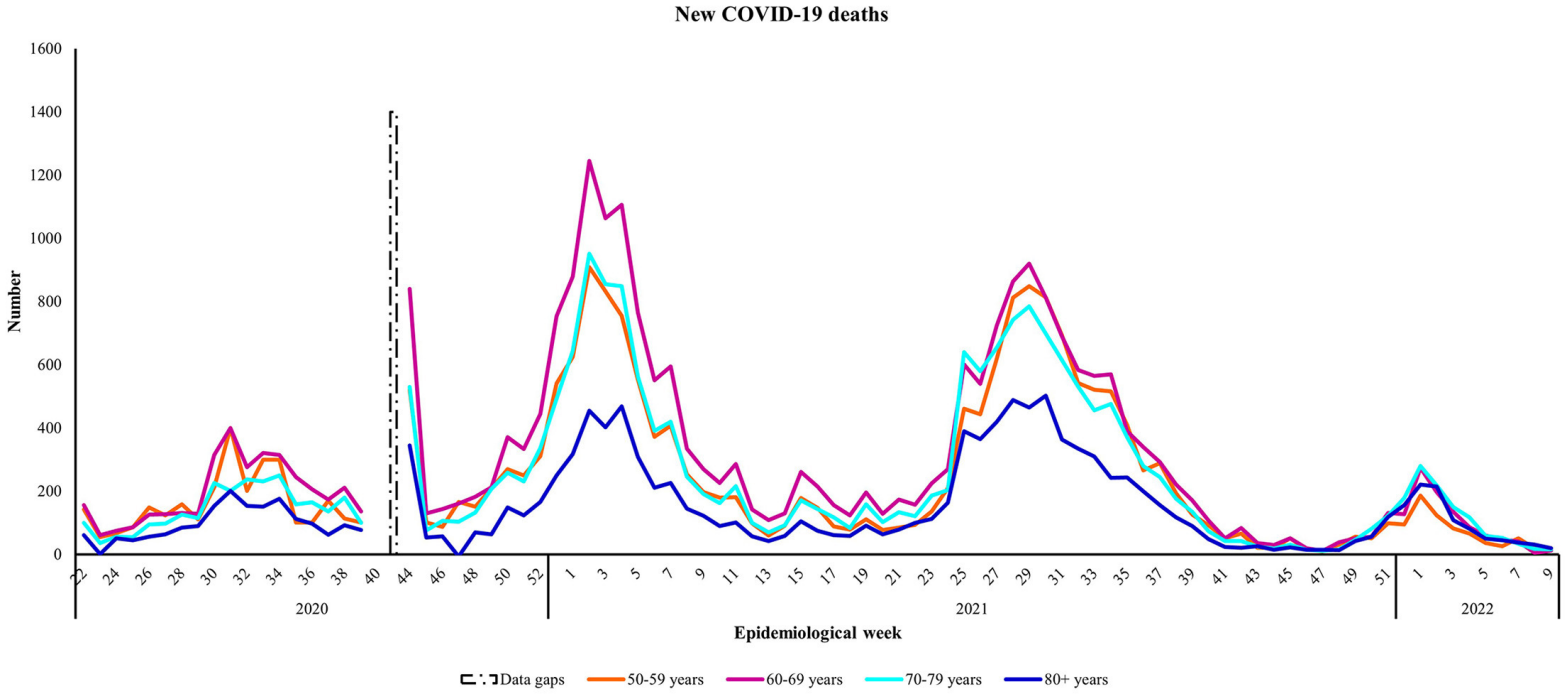
The number of new COVID-19 deaths by age group from May 30th, 2020 to March 5th, 2022, South Africa. Data source: https://www.nicd.ac.za/diseases-a-z-index/disease-index-covid-19/surveillance-reports/daily-hospital-surveillance-datcov-report/.

**Figure 5:**
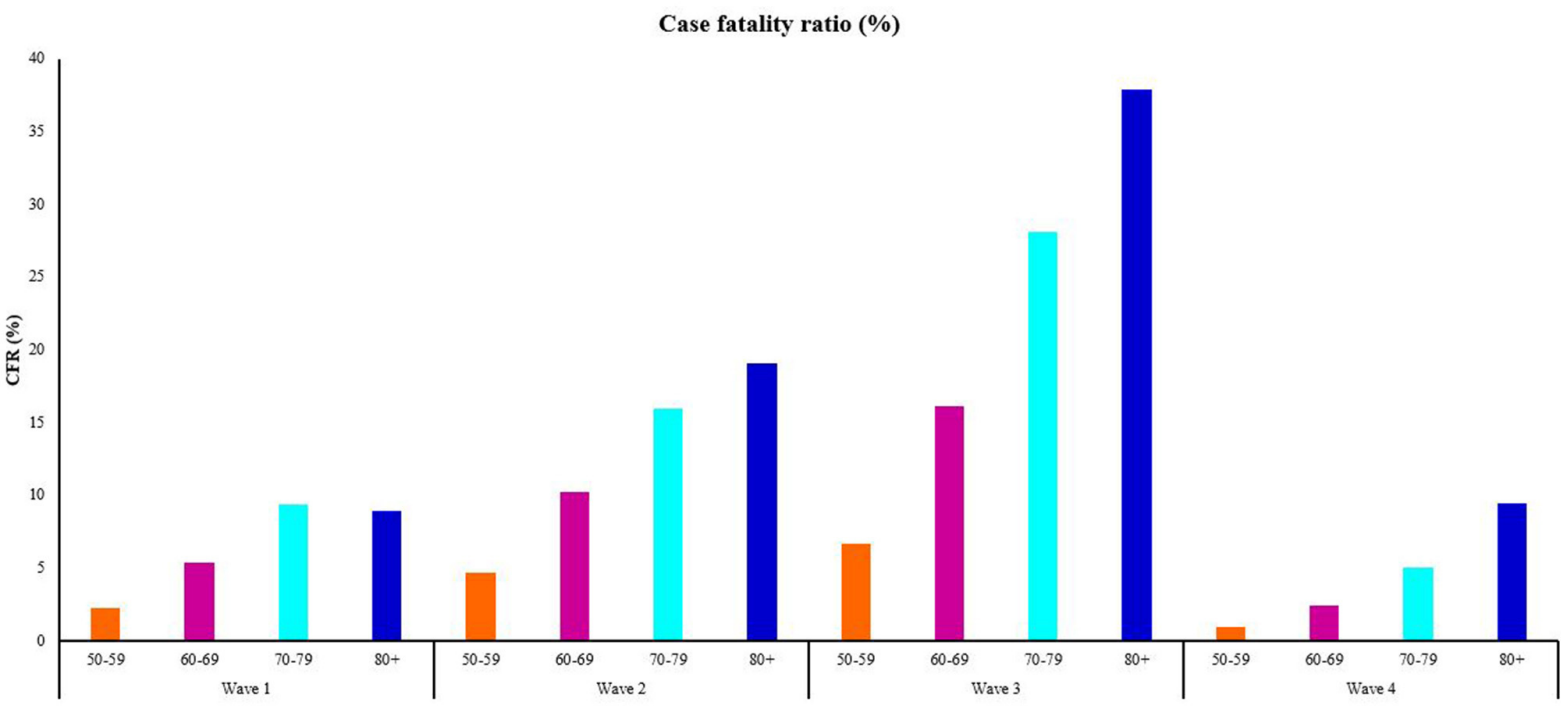
The proportion of individuals within each age group who died from the total number of confirmed cases, by COVID-19 wave from May 30th, 2020 to March 5th, 2022, South Africa. Data source: https://www.nicd.ac.za/diseases-a-z-index/disease-index-covid-19/surveillance-reports/daily-hospital-surveillance-datcov-report/.

Analyses of the number of new COVID-19 deaths (Figure 4) among older persons showed similarities between the 50–59 years and 70–79 year old age groups. The average observed number of COVID-19 deaths was higher among people aged 60–69 years (*N* = 4,999), compared to people aged 50–59 years (*N* = 4,108) and 70–79 years (*N* = 4,134) (Table 2). People aged 50–59, 60–69, and 70–79 years were most affected during Wave Two, whereas a higher observed number of new deaths occurred during Wave Three among people aged ≥ 80 years. When comparing the case fatality ratios among the target age groups, across the waves, people aged ≥ 80 years showed the greatest vulnerability, especially during Wave Three (Figure 5). The exception was in Wave One, where a higher case fatality ratio was seen among people aged 70–79 years. As with the hospitalizations, age-specific death rates increased with increasing age, showing an almost 5-fold increase between people aged 50–59 and ≥80 years in Wave Three (Table 2). The average age-specific mortality rates across all four epidemic waves suggests that it was mostly older persons aged ≥ 70 years that succumbed to COVID-19 over the 2-year study period.

### 4.5. Vaccination

**Figure 6:**
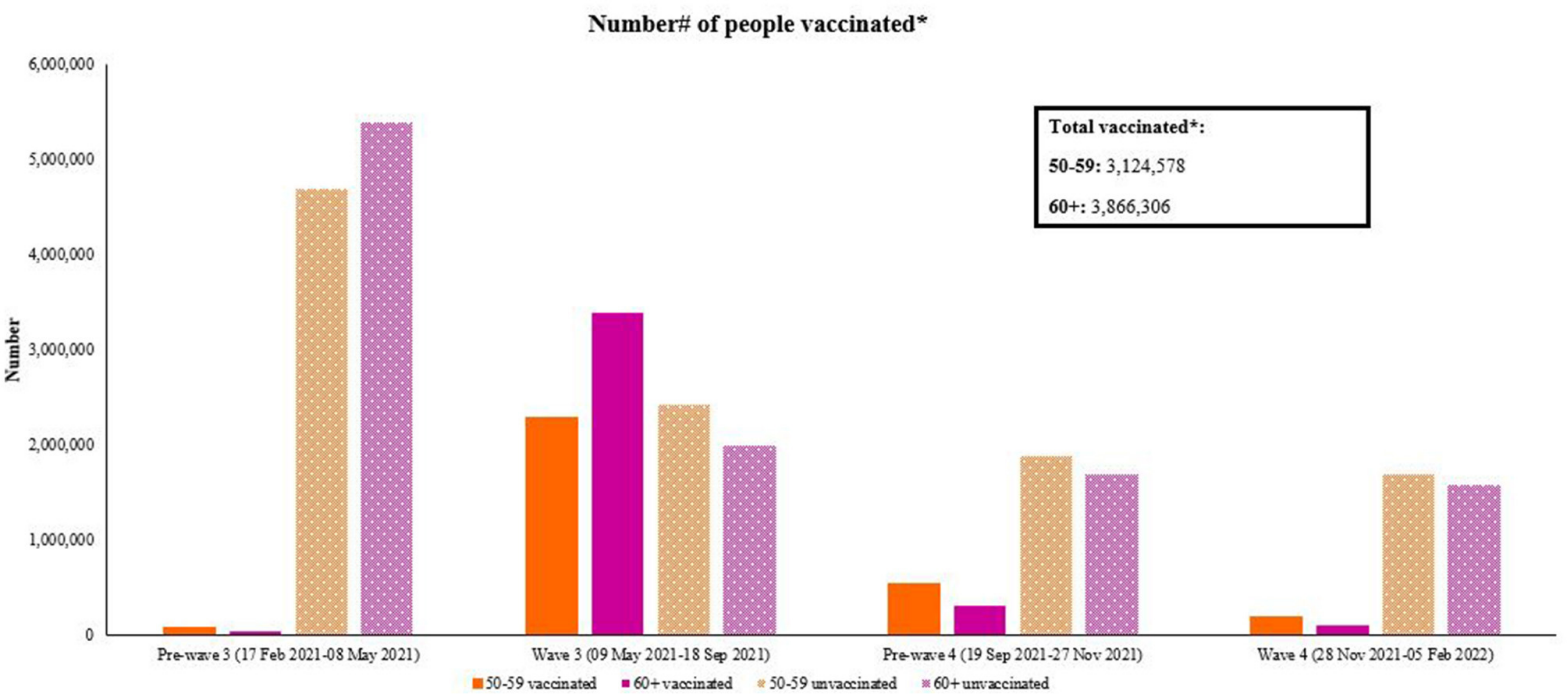
Number^#^ of 50–59 and ≥60 years vaccinated within each wave period. *Includes first Johnson & Johnson and first Pfizer dose. ^**#**^Vaccination numbers were subtracted for each wave period from the total cumulative vaccinations administered, for each age group. Data source: https://sacoronavirus.co.za/vaccine-updates/.

Vaccination numbers were subtracted for each wave period from the total cumulative vaccinations administered, for the near-old and older persons age group (Figure 6). When vaccines were rolled out pre-Wave Three, vaccine uptake was higher among people aged 50–59 years (*N* = 83,149) than people ≥ 60 years (*N* = 35,169) in terms of absolute numbers. This is likely due to the phased national rollout strategy which began with healthcare workers and then rolled out to people aged ≥ 60 years from May 17th, 2021 (epidemiological week 20 of 2021). In terms of age-specific rates, Table 2 shows that uptake was indeed higher among those ≥60 years compared to people aged 50–59 years both in terms of absolute numbers and rates, during the first vaccination roll out phase. Prior to and during epidemic Wave Four, vaccine uptake among people aged 50–59 years was higher (*N* = 363,000) than uptake among people aged ≥ 60 years (*N* = 207,000). Age-specific rates for people who took a first dose vaccine after Wave Three and up to and during Wave Four was almost double among near-old people aged 50–59 years compared to older persons aged ≥ 60 years. However, inspection of the vaccine naïve numbers for both age groups show they were similar for people aged 50–59 years and ≥60 years after Wave Three, suggesting consistent gaps and stagnation in vaccine uptake for both age groups.

## 5. Discussion

Studying heterogeneity of COVID-19 among near-old and older persons is a crucial step toward identifying their risk level and proposing timely interventions, toward safeguarding their health and wellbeing. Thus, the study examined the growth and trajectory of the epidemic within discrete near-old and older persons age categories over 2 years in South Africa using absolute numbers extracted from freely accessible epidemiological data and age-specific rates for each age group. During this time period, South Africa adopted five lockdown alert levels as part of a risk adjusted strategy from March 27th, 2020 until April 5th, 2022 and some advisories were specific to older persons. During lockdown levels four and five, all people aged ≥ 60 years and those with underlying medical conditions were advised to stay at home unless under exceptional circumstances. As the lockdown level restrictions were relaxed, employers were advised to adopt measures to cater for employees aged ≥ 60 years.

Our key findings based on age-specific epidemiological data show different profiles for near-old and older persons over the first four epidemic waves covering a 2-year period in South Africa. Age-related differentials emerged when comparing COVID-19 surveillance outcomes in terms of absolute numbers (cases, hospitalizations, and deaths) between the near-old (50–59 years) and older persons (≥60 years). For instance, a higher burden of COVID-19 confirmed cases, deaths and hospital admissions were observed in people aged 50–59 and 60–69 years, representing the younger of our target age groups. The age-specific case rates suggests that people aged 50–59 years and ≥80 years were most susceptible to acquiring COVID-19 over the 2-year study period. However, severity of outcomes relating to hospital admissions and deaths increased with age.

Another study using data from South Africa, reported COVID-19 hospitalizations and mortality for the period March 2020 to January 2021 (i.e., covering Waves One and Two) and found that most hospitalizations occurred among people aged 50–59 years (22.5%) and 60–69 years (18.9%) (total sample of hospitalizations (*N* = 215,028) compared to other age groups (15). Similarly, findings using DATCOV data from March 2020 to March 2021, showed that age was the strongest predictor of mortality among hospitalizations (median age of hospitalizations was 54 years) (28). It also noted moderate increased mortality risk among people living with HIV who were not on antiretroviral therapy and among people who had tuberculosis (28).

Our analyses of population adjusted age-specific hospitalization rates over a 2-year study period suggests that, overall, older persons aged ≥ 70 years were most susceptible to infections requiring hospitalization, compared to their younger counterparts. There was an overall trend of increasing confirmed cases and hospitalizations during South Africa's first three epidemic waves (June 2020 to September 2021) for all ages but the majority of hospitalizations with a known outcome and severe disease were reported among people aged ≥ 60 years during Waves Two (Beta variant dominant) and Three (Delta variant dominant) (17).

Similar to the hospitalizations, the age-specific death rates across all four epidemic waves examined, suggests that older persons aged ≥ 70 years were most likely to succumb to COVID-19 over the 2-year study period. It must be noted that the mortality estimates do not account for excess deaths. Our findings are consistent with surveillance data reported early in the pandemic, which established that the median age of confirmed COVID-19 cases was 51 years and fatality rates for people over 80 years of age were estimated to be five times higher than the global average as at April 2020 (11, 29). Similar patterns have been observed in all parts of the world, with most fatalities occurring among people aged ≥ 60 years in developed countries (11). A review of data from residential care homes within 22 non-African countries as at February 2021, reported substantial mortality. There were over 300,000 care home deaths, which accounted for 41% of the total COVID-19 deaths in the general population of the countries with available (30). Most excess deaths in South Africa occurred among people aged 60 years and older between 2020 and 2021 (2). Data show the number of 2021 deaths in South Africa approximated the number of deaths at the height of the HIV/AIDS epidemic in the country, whereby ~702,000 deaths were reported in 2006 and 701,000 deaths were reported in 2021 (31).

At a population level, several African countries have lower life expectancies and elevated levels of pre-existing conditions, comorbidities and living circumstances. There are also biological mechanisms relating to the aging immune system which impact on their quality of life and wellbeing (19). A decline in life expectancy since the emergence of COVID-19 was also reported for South Africa, with the estimated total life expectancy at birth dropping from 64.7 years in 2019 (27) to 61.7 years in 2021 (2), followed by a slight increase to 62.8 years in 2022 (31). COVID-19 infection can result in illness that is either mild with short recovery periods in some people, or life-threatening or causing long-term consequences in others (19). This suggests that age as a risk factor is multi-dimensional and needs continued monitoring and surveillance of infections and outcomes, together with long-term studies among near-old and older persons.

With the spread of COVID-19, there was a need for rapid mobilization of resources to gather and collate surveillance data for monitoring purposes and several African countries used insights from their surveillance systems (32). In early March 2020, the NICD in South Africa declared COVID-19 a category one notifiable medical condition (33). Recently researchers gave a detailed account of the epidemiological surveillance systems that were developed and used to inform South Africa's epidemic response (24). Briefly, the NICD was the initial referral laboratory where samples were sent for diagnostic testing. With community transmission and subsequent demand for testing increasing rapidly in 2020, the NHLS and private sector laboratories also conducted diagnostic testing using reverse transcriptase real-time PCR, which detects SARS-CoV-2 viral genetic material. Rapid antigen-based tests were implemented in November 2020 (34). Testing was widely expanded to people meeting the case definition for testing. It must be noted that the case definition for testing was updated several times. Data systems were promptly implemented to capture laboratory test data and monitor confirmed cases as part of the NMCSS (24).

Initial strategies by way of rapid mobilization to understand the epidemic in South Africa included community screening and testing where healthcare workers were deployed into communities to test people and their contacts and refer them to health facilities (35). This was in addition to contact tracing, where people identified as positive were contacted to determine close contacts and whether or not they were symptomatic. This strategy, however, faced several challenges, mostly due to a small number of people identified and poor uptake of digital technologies (COVID Connect self-services). Recognizing the risk for older persons particularly in congregate settings, the NICD had expanded surveillance to care facilities in South Africa in June 2020 and the positivity rate among residents and staff was 10% (*N* = 4,825) from 19 facilities by August 2020 (36). The South African COVID-19 country report also highlighted the heightened risk of people living in long-term care facilities (37).

These methods, although useful in the initial stages of the epidemic, became difficult to implement as the number of people who tested positive and exposed increased. This is also difficult in resource-strained settings, where human capacity to perform screening and contact tracing is limited. The NICD/NHLS noted the limitations related to testing. These include backlogs in laboratory processing particularly during high transmission periods, prioritization of severe cases for testing would introduce bias toward the number of people testing positive and periodic stock outs of consumables (32, 33). Researchers have described the test positivity as a useful metric when asymptomatic cases are high, and this, together with plots of the number of active confirmed cases, can be used to monitor the epidemic (38). However, as lockdown restrictions have been lifted and severity of COVID-19 has decreased, the number of people testing has declined (39).

Vaccines were made available in South Africa before the spike in cases reflecting the Third COVID-19 Wave occurred, which was driven by the Delta variant in South Africa. Vaccine rollouts in South Africa were based on a three-phased approach, to target those with higher contact and risk of severe COVID-19 infection, including healthcare workers, educators, those with co-morbidities and older persons aged 60 years and older. Hence efforts were concentrated initially toward getting vulnerable and highly exposed populations vaccinated timeously. A mixed methods community-based study conducted in South Africa during June-July 2021 in three high COVID-19 burden provinces found that 20% (*N* = 1,193) of respondents over 55 years were hesitant to receive a COVID-19 vaccine once it became available (40). Analyses of a longitudinal telephonic National Income Dynamics Study among adults in South Africa where older persons aged ≥ 60 comprised ~14% of the sample (~5,000 people from their survey waves four/five), found that willingness to get a COVID-19 vaccine significantly increased over time (41). However, approximately one quarter of the adult population in South Africa still expressed vaccine hesitancy (41) and willingness to take a vaccine may not translate into actual uptake. Lessons learnt from the Sisonke vaccination study among health care workers in South Africa were that consistent messaging and advocacy for vaccinations are crucial to mitigate hesitancy (42). Similarly, in the community-based study in South Africa, the recommendation was to address concerns about vaccine safety, whilst acknowledging that personal motivation and social influences also play a part in vaccine uptake or hesitancy (40). Vaccination has been shown to be effective in reducing severe disease and death, however, vaccination rates and uptake of booster shots might stagnate in view of new variants resulting in less severe outcomes, as well as a move globally toward a “pre-pandemic” world.

Beyond morbidity and mortality, it is widely documented that the challenges related to the COVID-19 pandemic among older people have had multiple interrelated implications for their health and wellbeing. While there are several biological factors which explain the variations among near-old and older people, they face additional social challenges such as social isolation, and loneliness. In the South African context, older people living in precarious situations may also face higher risks of violence, abuse, and neglect than other groups. In addition, some may not have access to health services, water and sanitation facilities, as well as other forms of support and assistance. Furthermore, older persons are likely caregivers within their families and often provide support for the sick, therefore increasing their risk of exposure to the virus. This is particularly true in contexts where health systems and long-term care provision are weak. Social networks tend to decline with aging, emanating from deteriorating health, mobility issues and death of family and friends. Older people may therefore have few close relationships or live alone (43). Environmental barriers also impact on social isolation and loneliness of older people, such as the feeling of being unsafe in their neighborhoods, especially in high crime contexts (44).

Age-specific COVID-19 related research for older people remains of critical importance and profound changes are needed in the way that research is designed and delivered for older people. However, this also presents new opportunities to deliver responses through research which can safeguard and protect near-old and older persons (45). This includes the need for data to be disaggregated at smaller age intervals (5- to 10-year bands) and for these data to be freely accessible to enable further work. The study findings have important implications. For instance, all people, including near-old and older people, have the right to equitable healthcare including timely access to health care and disease risk information. Strategies should accommodate the additional risks to near-old and older people due to their living and social circumstances, and the needs of each distinct age category. As other authors pointed out, in the next phase of the epidemic response, there is a need to target higher risk congregated settings together with prevalence studies to supplement existing information (32, 46). There is also a need for augmentation and integration of surveillance systems in African countries, together with the use of digital innovations for rapid detection and response to outbreaks and other epidemics (46).

The COVID-19 pandemic has exposed societal ageism and social ills including violence, abuse and neglect (9, 47). Highlighting the heterogeneity of issues pertaining to near-old and older persons offers an important opportunity for age-inclusive and age-appropriate targeted services and interventions for epidemic and outbreak preparedness. The impact of the pandemic on near-old and older people is now well recognized. However, there is need for nuanced approaches to and interventions for older persons, as COVID-19 transitions to becomes becoming endemic in South Africa.

## 6. Conclusion and recommendations

Moving from risk-adjusted strategies emphasizing epidemic control to lifting of COVID-19 restrictions and non-pharmaceutical interventions in the general population, specific strategies are needed for health promotion messaging accounting for heterogeneity (different risk profiles for acquisition, hospitalization, and deaths) among near-old and older persons. Near-old persons represent a group transitioning into older persons. Clinical management of confirmed COVID-19 cases evolved rapidly hence prompt health-seeking behavior should be encouraged, including testing and diagnosis, particularly for those high-risk older persons with comorbidities, to mitigate onset of severe illness or complications. This is especially important in congregate settings such as residential facilities for older persons, or in venues where they receive social care and support for health-related issues. Understanding the factors driving vaccination hesitancy in our target age groups who choose not to vaccinate or receive booster doses is needed. Risk tools and e-health for near-old and older persons should also be developed and made accessible given their technological capacities and challenges, as countries move from a national population-level risk approach to an individual-level risk approach, where the individual takes responsibility for their own risk adjusted strategy to preserve their health and wellbeing.

## 7. Limitations

Readily accessible disaggregated data were used to look at near-old and older persons age groups from reports and dashboards and were manually extracted. Thus, the data had to be synthesized and processed for the analyses. Gaps in the data by age had to be removed in the analyses. In addition, testing data were not available for the specific target age groups. Health-seeking behavior differs by age and sex for other health issues, which might contribute to observed differences in the cumulative and new case counts between age groups. This study did not analyze potential risk factors for the observed outcomes for the age groups, due to the lack of individual-level data. Nevertheless, the analyses presented show the utility of using publicly available surveillance data to describe the epidemiological trends of COVID-19 among older persons over a 2-year period to supplement efforts highlighting their specific needs.

## Data availability statement

Publicly available datasets were analyzed in this study. These data can be found here: https://www.nicd.ac.za/diseases-a-z-index/disease-index-covid-19/surveillance-reports/weekly-testing-summary/; https://www.nicd.ac.za/diseases-a-z-index/disease-index-covid-19/surveillance-reports/weekly-epidemiological-brief/; https://www.nicd.ac.za/diseases-a-z-index/disease-index-covid-19/surveillance-reports/daily-hospital-surveillance-datcov-report/; https://www.nicd.ac.za/diseases-a-z-index/disease-index-covid-19/surveillance-reports/daily-hospital-surveillance-datcov-report/; https://sacoronavirus.co.za/vaccine-updates/.

## Ethics statement

The study was approved by the Human Sciences Research Council Ethics Committee (REC) Protocol Number: (REC 3/17/03/21). Written informed consent for participation was not required for this study in accordance with the national legislation and the institutional requirements.

## Author contributions

NA and IN conceptualized the paper. NA, IN, MM, and SD extracted and analyzed the data. NA, IN, and SD drafted the paper. All authors critically reviewed and revised the manuscript and approved it for submission.
